# Supplementary material for: Knowledge mobilisation: an exploratory qualitative interview study to confirm and envision modification of lay and practitioner eczema mindlines to improve consultation experiences and self-management in primary care in the UK
Source: BMJ Open. 2019 Jun 28;9(6):e028225. doi: 10.1136/bmjopen-2018-028225 (PMC6609068; doi:10.1136/bmjopen-2018-028225)
Supplement: Author's manuscript [file bmjopen-2018-028225.draft_revisions.pdf]

BMJ Open

BMJ Open is committed to open peer review. As part of this commitment we make the peer review history of every article we publish publicly available.

When an article is published we post the peer reviewers' comments and the authors' responses online. We also post the versions of the paper that were used during peer review. These are the versions that the peer review comments apply to.

The versions of the paper that follow are the versions that were submitted during the peer review process. They are not the versions of record or the final published versions. They should not be cited or distributed as the published version of this manuscript.

BMJ Open is an open access journal and the full, final, typeset and author-corrected version of record of the manuscript is available on our site with no access controls, subscription charges or pay-per-view fees (http://bmjopen.bmj.com).

If you have any questions on BMJ Open's open peer review process please email info.bmjopen@bmj.com

BMJ Open

Knowledge mobilisation: an exploratory qualitative interview study to confirm and envision modification of lay and practitioner eczema mindlines to improve consultation experiences and self-management in primary care in the United Kingdom

Journal:	*BMJ Open*
Manuscript ID	bmjopen-2018-028225
Article Type:	Research
Date Submitted by the Author:	27-Nov-2018
Complete List of Authors:	Cowdell, Fiona; Birmingham City University, Faculty of Health Education and Life Sciences
Keywords:	Mindlines, Knowledge mobilisation, Eczema < DERMATOLOGY, PRIMARY CARE, Self-management, Consultation experiences

SCHOLARONE™
Manuscripts

BMJ Open Mindline confirmation submitted 27.11.18

Title Page

Article title: Knowledge mobilisation: an exploratory qualitative interview study to confirm and envision modification of lay and practitioner eczema mindlines to improve consultation experiences and self-management in primary care in the United Kingdom

Author information:
Fiona Cowdell
DProf, RN
Professor of Nursing and Health Research
Faculty of Health, Education and Life Sciences
Birmingham City University
220 Ravensbury House
Westbourne Road
Edgbaston
Birmingham
B15 3TN
UK
Telephone: +44 (0)121 300 4345
Email: Fiona.cowdell@bcu.ac.uk
orcid.org/0000-0002-9355-8059

Word count: 4265

Number of figures and tables: Figures 2, Tables 4

Key words: *Ba*, consultation, eczema, knowledge mobilisation, mindlines, primary care, self-management, qualitative

Disclaimer and source of support:
This article presents independent research funded by the National Institute for Health Research (NIHR). The views expressed are those of the author and not necessarily those of the NHS, the NIHR or the Department of Health.

For peer review only - http://bmjopen.bmj.com/site/about/guidelines.xhtml

BMJ Open Mindline confirmation submitted 27.11.18

Knowledge mobilisation: an exploratory qualitative interview study to confirm and envision modification of lay and practitioner eczema mindlines to improve consultation experiences and self-management in primary care in the United Kingdom

Abstract

Objectives: To investigate whether initial eczema mindlines, "collectively reinforced, internalised, tacit guidelines", are an accurate representation of the experiences of lay people and practitioners in primary care and to explore how these mindlines may best be revised to improve eczema care.

Design: Exploratory qualitative interviews with constant comparative data analysis.

Setting: United Kingdom, primary care

Participants: People with eczema or parents of children with eczema (n=19) and primary care practitioners (n=13).

Results: Interview data were analysed using constant comparison of new data with existing initial eczema mindlines to identify areas of agreement and disagreement. Data were mined for participant's thoughts about *whose* mindlines should be modified, *how* this may be achieved and *what* core content is essential. Eczema mindlines and the spiral of knowledge creation, from which they evolved, intuitively made sense. Participants offered examples of how their eczema knowledge is continually produced and transformed as they interact with others. They reported diverse and wide-ranging influences on their thinking and recognised the critical relationship between lay and practitioner mindlines. For this reason they advocated modifying lay and practitioner mindlines in parallel. Participants advised amendment based on consistent information directed to all who influence eczema care. They stipulated information should come from trusted sources and be easy to access, distilled, practical, contextually relevant and amenable to assimilation.

Conclusions: The purpose of this work is improve primary care consultation experiences and self-management in eczema. The remaining challenge is to find novel, simple and pragmatic methods of modifying eczema mindlines to instill shared and consistent understanding. Given the prevalence of eczema and the scope of people who influence self-care, interventions should transcend patient-practitioner boundaries and address the wider community. One conceptually congruent approach is to create a *Ba*, which in this case would be a virtual space for generating and sharing eczema knowledge.

For peer review only - http://bmjopen.bmj.com/site/about/guidelines.xhtml

BMJ Open Mindline confirmation submitted 27.11.18

Strengths and limitations

- Novel study confirming and extending existing lay and practitioner eczema mindlines
- Diverse sample of UK patients and parents of children with eczema and primary care practitioners
- Enhanced understanding of *whose* mindlines should be modified and *when* and *how* offers a starting point for the complex process of mindline amendment.

For peer review only

For peer review only - http://bmjopen.bmj.com/site/about/guidelines.xhtml

Introduction

Eczema is a common and burdensome long-term skin condition (1) predominantly treated in primary care (2). People seek advice from general practitioners (GPs), nurses, health visitors and pharmacy staff. Consultations with GPs can be frustrating for both patient (3) and practitioner (4, 5) and there may be considerable differences between beliefs about assessment and treatment (6). Nurse consultations tend to be more positively regarded (7, 8). Research into the role of pharmacy staff is limited (9).

The essence of eczema treatment in primary care is the regular application of emollients, at least daily and often for many years, with or without intermittent use of topical corticosteroids (TCS). Non-adherence is common due to high self-management demand (10) and limited, sometimes conflicting, information (11). Effective self-management can improve disease outcomes and quality of life (12). Interventions to support eczema self-management are poorly understood, are not available to all, can be resource intensive and have varying impact (13). Eczema is not formally categorised as a long-term condition (14).

Knowledge mobilisation (KM) is concerned with the creation and movement of new knowledge to where it can be most useful (15). To be effective KM activity must be relational, constructed from social interaction and context-specific (16-18). One method of mobilising knowledge is amending mindlines. Mindlines were developed from the work of Polyani (19) and Nonaka & Takeuchi (20) specifically the concept of the **S**ocialization, **E**xternalization, **C**ombination, **I**nternalization (SECI) spiral (17) in which knowledge is perpetually produced and transformed as users interact, collaborate and learn (37). Mindlines are "collectively reinforced, internalised tacit guidelines". They lie beneath clinical decision-making (21) and are underpinned by acceptance that tacit knowledge is a more powerful influencer of action than formal codified knowledge (19, 20). Mindlines are predicated on the notion of a flexible, embodied and intersubjective understanding of knowledge, the existence of multiple realties and acceptance of knowledge being context-specific (17). In their seminal work Gabbay and LeMay identified mindlines amongst practitioners in primary care (21); this and subsequent work has been frequently cited but minimal investigation has been undertaken on condition specific mindlines or the existence of the lay equivalent of mindlines (22).

A recent ethnographic study investigated the construction of healthcare practitioner and lay eczema mindlines in primary care. From this initial models of eczema mindlines were developed. For practitioners eczema was a low priority condition for which the "*recipe doesn't change".* Their

For peer review only - http://bmjopen.bmj.com/site/about/guidelines.xhtml

mindlines were built on brief and limited early career education, general and focused internet searches, local formularies and guidance, tacit knowledge and interactions with patients and, to some extent, other practitioners. Differences in mindline development were noted between professions (23). On the whole they perceived limited need to update their eczema knowledge. The initial model of lay (person with eczema or caring for a child with the condition) mindlines indicated that, like practitioners, their knowledge was built up over time from multiple sources. They comprise interactions with family, friends and the wider community and with practitioners, general and focused internet searches, media coverage, trial and error and tacit knowledge. The desire for knowledge was most marked when people were self-managing by default, those who had become disillusioned and had disengaged from primary care (3).

The purpose of the study reported here is to: (i) investigate whether the initial practitioner and lay mindlines are an accurate representation of the realities experienced by practitioners and lay people in primary care and (ii) consider how eczema mindlines may best be revised or modified by adding reliable and useful knowledge and by erasing outdated or inaccurate information thus potentially improve quality of eczema consultations and self-management.

Methods

Aims

To: (i) investigate whether initial eczema mindlines are an accurate representation of the realities experienced by practitioners and lay people in primary care and (ii) consider how they may best be revised or modified.

Design

Exploratory qualitative interviews (24) with constant comparative data analysis (25).

Setting, participants and process

Data were collected by the author, a nurse and researcher, from December 2017-May 2018. Practitioners were recruited via the local Clinical Research Network and word of mouth and lay participants though a higher education institute website with mass sharing via word of mouth and media posts in an effort to recruit hard-to-reach groups who may not realise their value in this type of study (26). Maximum variation purposive sampling was applied (27). Single, individual, semi-structured, audio-taped interviews using a topic guide (table 1) were conducted with lay people (n=19, table 2) and practitioners with between one and 26 years clinical experience (n=13, table 3)

For peer review only - http://bmjopen.bmj.com/site/about/guidelines.xhtml

and lasted from 16 – 55 minutes. Interviews continued to the point of data sufficiency (28). Most interviews were face-to-face, in the participant's workplace, and a small number via telephone. In preparation, participants were sent a brief explanation of mindlines and illustrations of the relevant initial eczema mindline (3, 23).

Data analysis

Data collection and analysis were iterative processes (29, 30). Audio-data was professionally transcribed and transcripts proof read against recordings for accuracy. Data were analysed using a constant comparison approach (25) assessing new data in relation to initial mindlines to identify areas of agreement and or disagreement. Data was also mined for participant's understandings about mindlines and how best they may be modified.

Reflexivity

A reflexive attitude was maintained throughout, acknowledging own subjectivity and positioning as a nurse and skin health researcher and the influence that this may have on the study (31).

Ethical issues

The study was approved by a University Ethics Committee. Written consent was taken for interviews and participants consented to publication of anonymised quotes.

Patient and public involvement

Lay people were involved in developing the research question and designing the study. Results will be communicated to participants in a brief summary.

Results

The results are presented in two sections; i) the extent to which eczema mindlines were perceived as being an accurate representation of realities, identifying individual similarities and differences and then areas of connection, synergy and dissonance between lay and practitioner mindlines and ii) a synthesis of *whose* mindlines should be amended with *what* information and *how* this could best be achieved.

Are lay eczema mindlines a reasonable representation of realities?

Lay participants reported that the concept of mindlines intuitively made sense and noted broad congruence in sources of knowledge and beliefs. They reviewed each element of the existing lay

For peer review only - http://bmjopen.bmj.com/site/about/guidelines.xhtml

eczema mindline, that is: interactions with family, friends and the wider community and with practitioners, general and focused internet searches, media coverage, trial and error and tacit knowledge within the context of being content to self-manage or accept practitioner management or self-managing by default. Lay participants spoke at length about the presented mindline and provided examples and nuanced insights into the how, why and when their mindlines had been constructed.

Interactions with family and friends and the wider community were experienced by all. The most influential information was advice from mothers. Others reported picking up "*snippets*" of information, sometimes unbidden "*I've had someone on the Tube recommending a moisturiser*" (Female, 22). Suggestions could be overwhelming *"they were telling me their regimes or oh try this cream. Everybody had a cream to recommend and it got to the point where I was using so many things, you didn't know what was working from what wasn't"* (Female, 27) and often unfounded *"hearsay ... 'oh well so and so tried this', 'oh I know someone who did this'"* (Female, 28). Advice from practitioners was sometimes externally validated with other lay people before making a decision about treatment use.

Participants mainly spoke about GP consultations which were frequently viewed as unsatisfactory. GPs were perceived as having limited expertise "*sometimes I get the impression that they're doing no more than you would by Googling it"* (Female, 34) and working on a trial and error basis "*it's generally a bit of chuck everything at the wall and see what sticks"* (Male, 36). In contrast to the initial mindline the pathway of knowledge between lay people and practitioners was perceived as one-way. People experienced their views and experiences as being undervalued and suggested that they were provided with standard, often unhelpful, information, "*I think doctors think everyone's stupid …… I know the advice is well meant but it's generalistic"* (Male, 56). Some mentioned power differentials as the cause of their expertise being dismissed "*I interact with them as equal but he couldn't have that"* (Male, 53).

Lay participants also consulted with nurses, pharmacy staff, health visitors and a physician's assistant. These meetings were more positively evaluated, for example after seeing a nurse "*I felt much more confident that they know in detail what they were dealing with …… they seemed to have a lot more knowledge about products"* (Female, 27). Pharmacists were another source of practical and personalised advice "*I saw the pharmacist at the practice she was actually very, very helpful, she*

For peer review only - http://bmjopen.bmj.com/site/about/guidelines.xhtml

came out with this keep the creams in the fridge, stroke them on ……… the two saviours were the pharmacists really" (Female, 75).

Most lay participants had searched the internet, the focus was invariably on finding new treatments or cure rather than understanding the condition. Most had used trusted sources such as NHS Choices, but for some this was "*a lovely set of generic blandishments"* (Male, 36). Many had "Googled" but were selective in using this information, "*lot of self-censoring, you know a filter*" (Female, 34). Finding reliable information electronically was challenging and some withdrew *"I'm wary of Google in case you get frightened by things that you don't need to see"* (Female, 34).

Other media sources, including leaflets, newspapers and magazines, television programmes and social media, were used to a lesser degree and also treated judiciously. Few people read patient information leaflets enclosed with medications describing them as of little practical value. Leaflets in GP surgeries and pharmacies were infrequently taken and poorly evaluated. A few read newspapers and magazines but were sceptical "*I'm quite an avid newsreader, periodically there are articles … don't always believe 100% because I think there could be something sponsored"* (Female, 39). Participants reported seeing occasional television programmes that influenced their thinking but not often their actions; the BBC was considered most trustworthy. Participant's valued social media for example Facebook and Twitter, the value here was perceived reality, "*a fellow sufferer saying 'this works for me*'" [Female, 34]. As with other knowledge sources participants filtered information, "*the critical eye that I have definitely makes me check and double check, are these valid sources and cross referenced?"* (Female, 26).

Trial and error was a familiar and frustrating experience for every lay participant "*you build up this medicine cabinet full of every ointment and then you have a set of stories about each one"* (Male, 53). Tacit knowledge was viewed as an amalgamation of personal experience accrued over time and hearsay from unknown or unremembered sources often from the wider community. Universal tacit knowledge was that TCS "*thin the skin*" and should be avoided as far as possible. No participants could trace how this thought had entered their mindline, "*I just grew up with that 'oh you have to be careful with this'"* (Female, children 13&15) and "*umm … I'm trying to think who told me*" (Female 34).

Participants identified with being content to self-manage or accept practitioner management or self-managing by default. Most described moving back and forth between these states, for example, at times of eczema flare, treatment failure or unsatisfactory consultations. These circumstances trigged

For peer review only - http://bmjopen.bmj.com/site/about/guidelines.xhtml

fresh attempts to find new ways of managing their eczema sometimes with clouded judgment, *"we just jump to unreliable sources or do irrational things because you can be tempted to, when you've got a really bad flare up"* (Female, 26).

Eczema mindlines instinctively made sense to lay participants and the content presented resonated with their thoughts, beliefs and experiences. They recognised the multiple influences, including the power of social interactions. Their analysis of personal eczema mindlines reinforced the complexity and difficulty in unravelling how all the elements coalesce particularly as, although content is similar for each individual, the way in which mindlines have developed over time had subtle variations. Lay participants linked efforts to amend mindlines closely with different phases of self-management and for this reason suggested that this should be integrated into the mindlines image (see figure 1).

Are practitioner eczema mindlines a reasonable representation of realities?

Practitioners agreed with the initial mindline but offered less discussion, for example "*so I think it's nice model actually, I think it's all fair, everything you've said is fair and reasonable and appropriate*" (Male GP). All agreed that eczema is not perceived as a priority in primary care, that treatment is limited by their local emollient formulary and that they exercised caution in prescribing TCS. A dominant influence on the mindlines of the four practitioners with self-reported expertise in eczema care was exposure to dermatology experts in secondary care. The trusted and structured guidance given had become firmly embedded leading to increased knowledge, skills and confidence and "*braver*" prescribing of non-formulary emollients (when clinically appropriate) and TCS. These experts contested the backdrop of low priority but concurred with the relative straightforwardness of treatment regimens whilst viewing these not as a "recipe" but as principles that should be individualised. The practitioner mindline illustration was amended for clarity (figure 2).

Mindline connections

Unprompted participants considered mindlines of the "other" and the interplay between these entities. Lay participants suggested a need for change in practitioner mindlines but focused on amending lay mindlines. Practitioners were certain about the best targets for mindline amendment. GPs stated that their own and lay mindlines should be amended but considered the former unlikely due to the low priority given to eczema. This view was reinforced by an expert nurse, "*GPs are entrenched in their views*" and a lay participant, "*it's a challenge with the entrenched stubbornness of the medical profession, people will not change their beliefs once they 'know something'*" (Lay

For peer review only - http://bmjopen.bmj.com/site/about/guidelines.xhtml

male, 36). Other practitioners advised that health visitors, nurses and community pharmacy staff should be targeted given their relative accessibility for patients.

A challenge for all participants was the volume of often contradictory information from a multitude of sources available to lay people. A GP recounted trying to persuade a patient to use TCS properly, "*I validate it with something and say, look I do extra work in dermatology, please just trust me, this is fine*". However his efforts were thwarted by the patient's existing mindlines and standard advice given by pharmacists which generated fear.

Practitioners almost universally identified the key reliable and useful knowledge that should be added to, and outdated or inaccurate information that should be erased from, existing mindlines. Core content is summarised in quotations from participants (table 4). Lay participants were less certain about the essential content but agreed key issues included understanding and accepting that eczema may be a long-term condition requiring ongoing treatment with emollients and that TCS have a place in the treatment of flares.

Synergy and dissonance between lay and practitioner mindlines

Returning to the original definition of mindlines as "collectively reinforced, internalised tacit guidelines" participants demonstrated both the complexity in development and the influential interplay between lay and practitioner mindlines. Participants identified areas of synergy and dissonance across and between mindlines. A key finding is that eczema knowledge does not involve just people living with the condition and practitioners but rather there is a pervasive social element, for example the received wisdom or "*old wives tale*" that TCSs "*thin the skin*" causing irreparable damage.

Perceived need to update was low among most practitioners and this pervaded some lay mindlines "*there's no point in wasting the doctors time*" (Male, 53). As a result eczema was sometimes relegated to an additional concern rather than the primary reason for consultation and then necessarily given limited attention by practitioners, thus reinforcing its low priority. Practitioners reported *"we don't often think about it as a chronic condition which needs regular follow up and a defined plan"* (Male, trainee GP), leading to reactive treatment with few follow-up appointments. Lack of maintenance therapy impacted on self-management leading to a cycle of flare management rather than a long-term, agreed management plan.

For peer review only - http://bmjopen.bmj.com/site/about/guidelines.xhtml

Mindline amendment

Mindline amendment proved a conundrum to all. Lay participants reflected on the futility of simply providing more information. Immediate suggestions including predictable approaches such as websites media and leaflets were made but rapidly dismissed. For example, *"website … but who would go on it?* (Male, 53) and "*leaflets in GP surgeries, but then you just get information fatigue*" (Male, 49). Similarly suggestions about using social media and 'apps' were voiced and rejected within the same sentence. Interactive fora were vetoed due to the need for leadership and moderation. One participant favoured a "*Facebook group specifically for eczema care, I'd probably be part of that ... good to have a safe place for people to talk*" (Female, 22) although she acknowledged that sustained participation was unlikely.

Pivotal factors in lay mindline amendment included "realness", exemplified by "*that's the stuff that I'm interested in and that I'll home in on are the forums where you've got people telling their stories*" (Male, 53) and "*people work off stories don't they, like, I'm living proof*" (Male, 36). Other people's stories were persuasive for some and disliked by others. Trustworthiness, ease of access and applicability to self were important, "*quick trusted and easy to access*" (Female, 34) from trusted sources "*pharmacies, a credible source of information*" (Female, children 17&20). All lay participants disliked the generic nature of current information "*eczema is very particular, people regard it as part of who they are, because it does shape what you're doing*" (Male, 53). Disillusionment and lack of motivation to change was identified as a challenge to mindline amendment. Interactions and relationships were addressed on several levels. Engagement was one hurdle "*you need a way of hooking people in, people who are likely to walk past and go 'I haven't got time to care about that'*" (Female, 34). The impact of social and relational factors suggested a need for wider amendment "*an effective way would be to normalise this thing, there's still a shame element around it, bringing it into normal conversations, everyday discussions, so it's not a thing that you're just hiding away*" (Female, 26). Shared, consistent information could be powerful in eradicating influential "*old wives tales*".

Practitioners proposed existing educational mechanisms for amending mindlines including: free taught sessions with "*really, really practical, emollient testing*" (Female, nurse) and information which can readily be applied in everyday practice; free online education; sessions during protected learning time. Practitioners rarely accessed available eczema education and were "*wary of messages from pharma or perceived messages from pharma*" (Female, GP). Expert practitioners were exasperated by the lack of motivation for change amongst generalist colleagues and the continued

For peer review only - http://bmjopen.bmj.com/site/about/guidelines.xhtml

provision “*outdated and wrong*” advice (Female, nurse). Practitioners were aware of existing patient-focused resources, for example NHS Choices, patient.co.uk, the Primary Care Dermatology Society and the British Association of Dermatologists Patient Information Leaflets, but rarely signposted or used these in consultations.

Participants pointed to the need for shared understandings and consistent advice through synergistic mindlines. Reflection on mindlines brought to the fore the diversity and magnitude of influencing factors and the extent to which knowledge was accessed in different circumstances, for example during an eczema flare. The need for consistency of information was a high priority and one best addressed by seeking to modify the multiple channels of information that contribute to lay and practitioner eczema mindlines in primary care with straightforward, evidence based messages.

Discussion

The first aim of this study was to investigate whether initial eczema mindlines are an accurate representation of the experiences of practitioners and lay people in primary care. Mindlines intuitively made sense to, and were confirmed by, all participants. Areas of synergy and dissonance were identified, as was the interplay between lay and practitioner eczema mindlines. Secondly mindline amendment was investigated. The need to modify and unify mindlines by adding reliable and useful knowledge and by erasing outdated or inaccurate information was universally agreed. However, strategies to achieve amendment proved elusive. The prevalence of eczema, the diversity of *how* and *when* mindlines are developed suggests a need to find focused strategies for wide-reaching, societal amendment to ensure simple, consistent messages to improve consultation experiences and self-management in primary care. Practitioners offered suggestions about essential areas for amendment.

This study is one of few to apply mindline theory to a specific condition and is original in identifying the relationship between lay and practitioner mindlines. The study conforms to the conventions of qualitative research in being rigorous (externally auditable thorough clear reporting), relevant (enhancing understanding of the subject), resonant with reader’s experiences and understandings and reflexive (31). The study has deepened understanding of *whose* mindlines should be modified and *when* and *how* this may best be achieved. This offers a robust starting point for the complex amendment process.

To date little attention has been given to the construction of lay (or patient) mindlines. Gabbay and LeMay (17) suggest a patient equivalent, but this possibility is poorly represented in other literature

For peer review only - http://bmjopen.bmj.com/site/about/guidelines.xhtml

(22). The term clientlines appears in one study (32) but is not fully explored. However, the notion that knowledge creation inevitably happens in patients is not contested (33) and is extended here in the breadth of influences on lay eczema mindlines. This could be attributable to prevalence, with most people knowing someone with eczema and the perception that it is a “health problem which is not an illness” (34) and therefore perceived as open to treatment suggestions from anyone.

Amendment of mindlines is rarely reported, a synthesis of 10 years of literature (n=340) identified 28 related to practitioner amendment. These emphasise the importance of collaborative learning, relationship building and effective leadership in the development of valid, collective, evidence-based mindlines (22). Repeating the search strategy used for this review in August 2018 revealed an abundance of new literature (n=422) but still little directly addressing how mindlines may best be amended. Allied literature indicates the need for a judicious approach to amending practitioner mindlines. Primary care practitioners are necessarily expert generalists (35) and have to know about many conditions. It is clearly impossible for any individual to process all available information. Practitioners use coping strategies such as “satisficing” that is, curtailing the amount of information gathered to enable them to make a “good enough” decision (36). Given the view that eczema is straightforward with treatment being standard and unchanging over the years, motivation to change practice is limited (23). Likewise treatment decisions are apparently made rapidly, perhaps fitting somewhere between fast, automatic, System 1 thinking and the more deliberative, conscious slow and effortful System 2 approach (37) and instead accessing knowledge from a well-worn path. The danger here is that *this* path may not lead to the best outcomes.

Mindlines evolved from the work of Polyani and Nonaka & Takeuchi, in particular, the SECI spiral (17) in which knowledge is perpetually produced and transformed as users interact, collaborate and learn (38). Gabbay and LeMay (21) point to the critical nature of knowledge-in-practice-in-context in which in each context new knowledge is converted by complex social processes. This current study concurs, finding that eczema knowledge is founded in a wider community (39, 40), sometimes facilitated by others (41) and often influenced by numerous, interacting personal and social factors (42). To date moves to improve eczema self-management and consultation experiences largely comprise educational and psychological interventions (13, 43). This study offers an alternative socially-mediated approach. Returning to the work of Nonaka offers new insights into mindline amendment drawing on the relationship between the SECI spiral and *Ba*. *Ba*, originally discussed by the Japanese philosopher Nishida (44, 45) and later refined by Shimizu (46), is a shared space for knowledge generation and sharing. *Ba* is not about the space per se (47), rather it is a shared

For peer review only - http://bmjopen.bmj.com/site/about/guidelines.xhtml

context-in-motion which advances over time and has no fixed boundaries or membership. It is about the here-and-now and thrives on people with different viewpoints (48). *Ba* space occurs naturally, is not prescribed, relationships are fluid, permeable and iterative (49, 50). It has four characteristics: originating (face-to-face, individual communications), interacting (face-to-face group dialogue), cyber (virtual group interactions) and exercising (virtual individual interactions) (50). In the main *Ba* has been considered in business organisations (51-56). More recently attention has been given to its application in other contexts, for example in making "big data" widely useful (57) and in supporting educational endeavours (50, 58, 59). To date there is no evidence of attempting to create *Ba* across lay-practitioner-community boundaries. Given the need to amend mindlines, both lay and practitioner, across the community the notion of deliberatively creating *Ba* and integrating innovative approaches to addressing each of the four characteristics in a specific geographic area offers a new opportunity for eczema knowledge mobilisation.

In some respects core treatment of eczema in primary care is relatively straightforward. Experts in the field propose "getting control" and "keeping control" (60) which requires emollient maintenance to reduce incidence and severity of flares and need for sufficient use of the right potency TCS when required. The key to effective self-management is supporting people to do these two things well and addressing the enormous challenges they face on a day-to-day enduring basis (61-64).

This study offers a new approach to improving consultation experiences and self-management through active and community-wide mindline amendment to develop consistent and shared understandings. Core reliable and useful knowledge that needs to be integrated, and outdated and inaccurate knowledge to be erased is identified. Mechanisms for change need to be socially-mediated, multi-faceted, tailored to specific groups and convey consistent, evidence based, core messages.

For peer review only - http://bmjopen.bmj.com/site/about/guidelines.xhtml

BMJ Open Mindline confirmation submitted 27.11.18

Contribution statement

FC is the sole contributor to this paper

Funding

This article presents independent research funded by the National Institute for Health Research (NIHR). The views expressed are those of the author and not necessarily those of the NHS, the NIHR or the Department of Health.

Ethical approval

The study was approved by a University Ethics Committee. Written consent was taken for interviews. Participants consented to publication of anonymised information.

Competing interests

None to declare

Acknowledgements

Thanks to Jay Nolan-Latchford for the mindline illustrations and to all the participants who shared their time and thoughts.

Data sharing statement

The datasets generated and/or analysed during the current study are not publicly available as they are not designed to be re-analysed by others but are available from the corresponding author on reasonable request.

For peer review only

BMJ Open Mindline confirmation submitted 27.11.18

References

1. Hay RJ, Johns NE, Williams HC, et al. The global burden of skin disease in 2010: an analysis of the prevalence and impact of skin conditions. J Invest Dermatol. 2014; 134: 1527-1534.
2. Schofield J, Grindlay D, Williams H, Skin conditions in the UK: a health care needs assessment. Centre of Evidence Based Dermatology, University of Nottingham; 2009.
3. Cowdell F. Knowledge mobilisation: an ethnographic study of the influence of lay mindlines on eczema self-management in primary care in the United Kingdom. BMJ Open. In press
4. Magin P, Adams J, Heading G, Pond C. Patients with skin disease and their relationships with their doctors: a qualitative study of patients with acne, psoriasis and eczema. Med J Aust. 2009; 190:62-64
5. Santer M, Burgess H, Yardley L, et al. Experiences of carers managing childhood eczema and their views on its treatment: A qualitative study. Br J Gen Pract. 2012; 62:261-267.
6. Powell K, Le Roux E, Banks J, et al. GP and parent dissonance about the assessment and treatment of childhood eczema in primary care: a qualitative study. BMJ Open. 2018; 8:e019633.
7. Nicol NH, Ersser SJ. The role of the nurse educator in managing atopic dermatitis. Immunol Allergy Clin. 2010; 30:369-383.
8. Rolinck-Werninghaus C, Trentmann M, Reich A, et al. Improved management of childhood atopic dermatitis after individually tailored nurse consultations: a pilot study. Pediat Allergy Immunol. 2015; 26: 805-810.
9. Tucker R, Stewart D. Why people seek advice from community pharmacies about skin problems. Int J Pharm Pract. 2015; 23(2): 150-153.
10. Santer M, Burgess H, Yardley L, et al. Managing childhood eczema: Qualitative study exploring carers' experiences of barriers and facilitators to treatment adherence. J Adv Nurs. *2013*; 69:2493-2501.
11. Teasdale EJ, Muller I, Santer M. 2017. Carers' views of topical corticosteroid use in childhood eczema: a qualitative study of online discussion forums. Br J Dermatol. 2017; 176(6):1500-1507.
12. Department of Health 2014 The Mandate: A mandate from the Government to NHS England: April 2014 – March 2015 https://www.gov.uk/government/uploads/system/uploads/attachment_data/file/383495/2902896_DoH_Mandate_Accessible_v0.2.pdf Accessed 4.7.18
13. Ridd MJ, King AJL, Le Roux E, et al. Systematic review of self-management interventions for people with eczema. Br J Dermatol. 2017; 177(3):719-734.
14. Hanlon P, Daines L, Campbell C. et al. Telehealth interventions to support self-management of long-term conditions: a systematic metareview of diabetes, heart failure, asthma, chronic obstructive pulmonary disease, and cancer. JMIR. 2017; 19:e172.
15. Ward V. 2017. Why, whose, what and how? A framework for knowledge mobilisers. Evid Policy. 2017; 13:477-497.
16. Ferlie E, Crilly T, Jashapara A, et al. Knowledge mobilisation in healthcare: a critical review of health sector and generic management literature. Soc Sci Med. 2012;74:1297-1304.
17. Gabbay J, Le May A. Practice-based evidence for healthcare: clinical mindlines. London: Routledge; 2010.
18. Plamondon K, Caxaj S. 2018. Toward Relational Practices for Enabling Knowledge-to-action in Health Systems: The Example of Deliberative Dialogue. Adv Nurs Sci. 2018; 41:18-29.
19. Polanyi M. The logic of tacit inference. Philos.1966; 41:1-18.
20. Nonaka I, Takeuchi H. The knowledge-creating company: How Japanese companies create the dynamics of innovation. Oxford: Oxford University Press; 1995.
21. Gabbay J, le May A. Evidence based guidelines or collectively constructed “mindlines?” Ethnographic study of knowledge management in primary care. BMJ. 2004; 329:1013.

For peer review only - http://bmjopen.bmj.com/site/about/guidelines.xhtml

BMJ Open Mindline confirmation submitted 27.11.18

22. Wieringa S, Greenhalgh T. 10 years of mindlines: a systematic review and commentary. Imp Sci. 2015; 10:45.
23. Cowdell Practitioner mindlines *awaiting editor decision with BMJ Open*
24. Stebbins RA. Exploratory research in the social sciences. Sage; 2001 May 14.
25. Cho JY, Lee EH. Reducing confusion about grounded theory and qualitative content analysis: Similarities and differences. The qualitative report. 2014;19(32):1-20.
26. Bonevski B, Randell M, Paul C, Chapman K, Twyman L, Bryant J, Brozek I, Hughes C. Reaching the hard-to-reach: a systematic review of strategies for improving health and medical research with socially disadvantaged groups. BMC Med Res Meth. 2014 Dec;14(1):42.
27. Tongco MD. Purposive sampling as a tool for informant selection. Ethnobotany Research and Applications. 2007. 31; 5:147-58.
28. O'Reilly M, Parker N. 'Unsatisfactory Saturation': a critical exploration of the notion of saturated sample sizes in qualitative research. Qual Res. 2013; 13:190-197.
29. Spradley JP. Participant observation. Illinois: Waveland Press; 2016.
30. Spradley JP. The ethnographic interview. Illinois: Waveland Press; 2016.
31. Finlay L. Phenomenology for psychotherapists: Researching the lived world. Oxford: Wiley Blackwell; 2011.
32. Chandler CI, Jones C, Boniface G et al. Guidelines and mindlines: why do clinical staff over-dignose malaria in Tanzania? A qualitative study. Malar J 2008;7:53
33. Launer J. Guidelines and mindlines. Postgraduate medical journal. 2015 Nov 1;91(1081):663-4.
34. Cornwell J. Hard-earned lives: accounts of health and illness from East London: Routledge Kegan & Paul;1984
35. Reeve J, Dowrick CF, Freeman GK, et al. Examining the practice of generalist expertise: a qualitative study identifying constraints and solutions. JRSM short reports. 2013; 4(12):2042533313510155.
36. Bate L, Hutchinson A, Underhill J, et al. How clinical decisions are made. Br J Clin Pharmacol. 2012; 74:614-20.
37. Kahneman D. Thinking, Fast and Slow. London: Penguin; 2011.
38. Nonaka I, Nishihara AH, Kawada H. Knowledge-based management theory. In Knowledge Creation in Public Administrations 2018 (pp. 1-21). Palgrave Macmillan, Cham.
39. Dobele A, Fry J, Rundle-Thiele S, Fry T. Caring for baby: what sources of information do mothers use and trust? Journal of Services Marketing. 2017 Oct 9;31(7):677-89.
40. Reifegerste D, Bachl M, Baumann E. Surrogate health information seeking in Europe: Influence of source type and social network variables. International journal of medical informatics. 2017 Jul 1;103:7-14.
41. Edwards M, Wood F, Davies M, Edwards A. 'Distributed health literacy': longitudinal qualitative analysis of the roles of health literacy mediators and social networks of people living with a long-term health condition. Health Expectations. 2015 Oct;18(5):1180-93.
42. Bigsby E, Hovick SR. Understanding associations between information seeking and scanning and health risk behaviors: An early test of the structural influence model. Health communication. 2018 Mar 4;33(3):315-25.
43. Ersser SJ, Cowdell F, Latter S, Gardiner E, Flohr C, Thompson AR, Jackson K, Farasat H, Ware F, Drury A. Psychological and educational interventions for atopic eczema in children. Cochrane Database of Systematic Reviews 2014, Issue 1. Art. No.: CD004054. DOI: 10.1002/14651858.CD004054.pub3.
44. Nishida K, Dilworth DA. Fundamental Problems of Philosophy the World of Action and the Dialectical World. Translated with an Introd. By David A. Dilworth.
45. Nishida K. An Inquiry into the Good, as translated by Abe, M. and C. Ives.(1990). New Haven./London: Yale University. 1921.
46. Shimizu H. Ba-principle: new logic for the real-time emergence of information. Holonics. 1995 Aug;5(1):67-79.

For peer review only - http://bmjopen.bmj.com/site/about/guidelines.xhtml

47. Bartolacci C, Cristalli C, Isidori D, Niccolini F. Ba virtual and inter-organizational evolution: a case study from a EU research project. Journal of Knowledge Management. 2016 Jul 11;20(4):793-811.
48. Nonaka I, Toyama R. The knowledge-creating theory revisited: knowledge creation as a synthesizing process. Knowledge management research & practice. 2003 Jul 1;1(1):2-10.
49. Nonaka I, Konno N. Methodology of knowledge creation. Tokyo: Toyo Keizai. 2003 Apr 17.
50. Krahe JA, Lalley C, Solomons NM. Beyond Survival: Fostering Growth and Innovation in Doctoral Study–A Concept Analysis of the Ba Space. International journal of nursing education scholarship. 2014 Jan 1;11(1):11-8.
51. Nonaka I, Von Krogh G, Voelpel S. Organizational knowledge creation theory: Evolutionary paths and future advances. Organization studies. 2006 Aug;27(8):1179-208.
52. Von Krogh G, Nonaka I, Rechsteiner L. Leadership in organizational knowledge creation: A review and framework. Journal of Management Studies. 2012 Jan 1;49(1):240-77.
53. Fayard PM. Strategic communities for knowledge creation: A Western proposal for the Japanese concept of Ba. Journal of Knowledge Management. 2003 Dec 1;7(5):25-31.
54. Corno F, Reinmoeller P, Nonaka I. Knowledge creation within industrial systems. Journal of management and governance. 1999 Dec 1;3(4):379-94.
55. Hussi T. Reconfiguring knowledge management–combining intellectual capital, intangible assets and knowledge creation. Journal of knowledge Management. 2004 Apr 1;8(2):36-52.
56. Tyagi S, Cai X, Yang K, Chambers T. Lean tools and methods to support efficient knowledge creation. International Journal of Information Management. 2015 Apr 1;35(2):204-14.
57. Philip J. An application of the dynamic knowledge creation model in big data. Technology in Society. 2018 Apr 5.
58. de Aguiar TR, Paterson AS. Sustainability on campus: knowledge creation through social and environmental reporting. Studies in Higher Education. 2017 Feb 22:1-3.
59. Gupta V, Jain N. Harnessing information and communication technologies for effective knowledge creation: Shaping the future of education. Journal of Enterprise Information Management. 2017 Sep 11;30(5):831-55.
60. Williams HC. Preventing eczema flares with topical corticosteroids or tacrolimus: which is best?. British Journal of Dermatology. 2011 Feb;164(2):231-3.
61. Santer M, Burgess H, Yardley L, et al., Managing childhood eczema: Qualitative study exploring carers' experiences of barriers and facilitators to treatment adherence. J Adv Nurs. 2013; 69:2493-2501.
62. Jackson K, Ersser SJ, Dennis H, et al., The eczema education programme: Intervention development and model feasibility. J Eur Acad Dermatol Venereol. 2014; 28:949-956.
63. Santer M, Muller I, Yardley L, et al. 'You don't know which bits to believe': Qualitative study exploring carers' experiences of seeking information on the internet about childhood eczema. BMJ Open. 2015; 5:e006339-e006339. doi:10.1136/bmjopen-2014-006339
64. Santer M, Muller I, Yardley L, et al. Parents' and carers' views about emollients for childhood eczema: Qualitative interview study. BMJ Open. *2016; 6:* e011887-e011887. doi:10.1136/bmjopen-2016-011887

For peer review only - http://bmjopen.bmj.com/site/about/guidelines.xhtml

BMJ Open Mindline confirmation submitted 27.11.18

Table 1: Interview topic guide

Table 2: Demographic details of lay interview participants

Table 3: Demographic details of practitioner interview participants

Table 4: Core eczema mindline content from practitioners

Figure 1: Lay eczema mindline

Revised lay eczema mindline

Figure 2: Practitioner eczema mindline

Revised practitioner eczema mindline

For peer review only

At the beginning of each interview the concept of mindlines and illustrations were discussed.<br>• How does this eczema mindline make sense to you?<br>• What are the similarities and differences in the way in which you know and think about eczema?<br>• How could eczema mindlines best be amended?<br>• Do you think there particular information that needs to be added or erased from existing mindlines?

Table 1: Interview topic guide

For peer review only

Self / child	Age	Gender
Self	28	Female
Self	36	Male
Child	6	Female
Self	53	Male
Self	34	Female
Children	13 and 15	Female
Self	75	Female
Self	26	Female
Self	49	Male
Self	28	Female
Self	39	Female
Child	5	Female
Self	24	Male
Child	7	Female
Children	17 and 20	Female
Self	22	Female
Self	56	Male
Self	38	Male
Self	48	Female

Table 2: Demographic details of lay interview participants

For peer review only

Role	Gender
Dermatology nurse in primary care	Female
Dermatology nurse in primary care	Female
Community pharmacist	Female
Trainee general practitioner	Male
Practice nurse	Female
Nurse practitioner	Male
General practitioner	Male
General practitioner	Male
General practitioner	Female
General practitioner	Male
General practitioner	Male
Practice nurse	Female
Practice nurse	Female

Table 3: Demographic details of practitioner interview participants

For peer review only

• *"never let anyone say to you again that eczema is just a bit of dry skin"*<br>• *"treat as a long term condition"*<br>• *"eczema needs maintenance treatment"*<br>• *"mantra of the best emollient is the one that [is used]"*<br>• *"don't be frightened [of] steroids and use them properly"*<br>• *"when it does flare up you've got to hit it hard quickly with your steroid"*

Table 4: Core eczema mindline content from practitioners

For peer review only

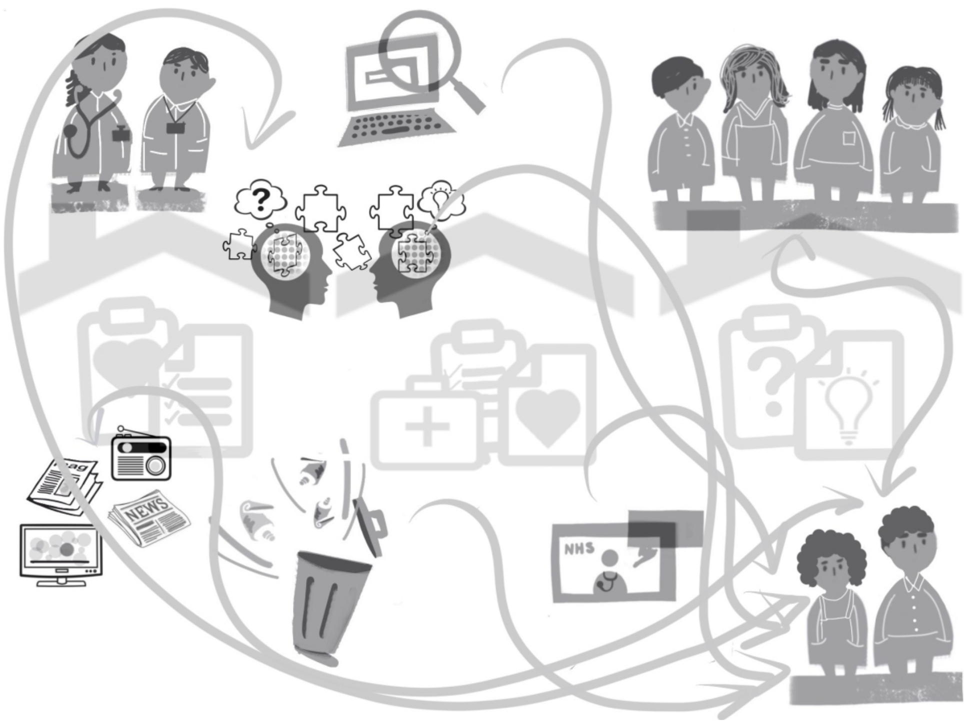

Lay eczema mindline

394x295mm (132 x 132 DPI)

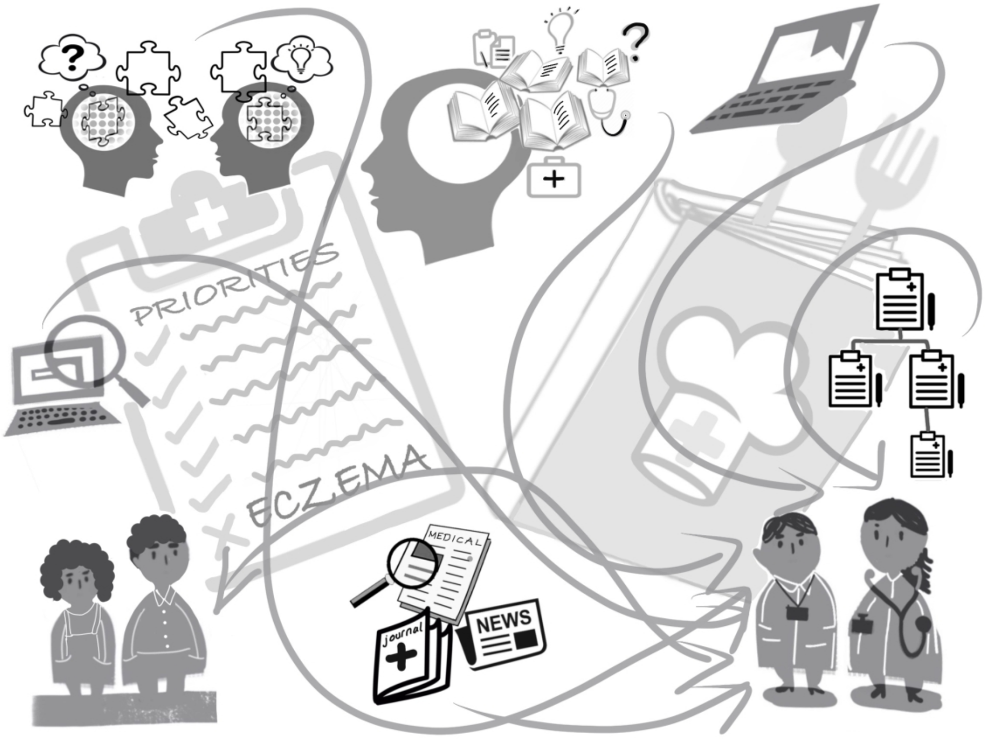

Practitioner eczema mindline

394x295mm (132 x 132 DPI)

BMJ Open Eczema mindlines COREQ 27.11.18

COREQ Statement

Knowledge mobilisation: an exploratory qualitative interview study to confirm and envision modification of lay and practitioner eczema mindlines to improve consultation experiences and self-management in primary care in the United Kingdom

Statement	Page no
**Domain 1: Research team and reflexivity**<br>*Personal Characteristics*<br>1. Interviewer/facilitator Which author/s conducted the interview or focus group?<br>*I conducted all observation and interviews*	5
2. Credentials What were the researcher's credentials? E.g. PhD, MD<br>*DProf, RN*	Title page
3. Occupation What was their occupation at the time of the study?<br>*Professor of Nursing and Health Research*	Title page
4. Gender Was the researcher male or female?<br>*Female*	Title page
5. Experience and training What experience or training did the researcher have?<br>*I have extensive experience in qualitative research.*	Title page
Relationship with participants<br>6. Relationship established Was a relationship established prior to study commencement?<br>*I established rapport with each participant at the time of interview.*	6
7. Participant knowledge of the interviewer<br>What did the participants know about the researcher? e.g. personal goals, reasons for doing the research<br>*I sent participants an information sheet prior to interviews and answered any questions, I reiterated this information at the beginning of each interview.*	6
8. Interviewer characteristics What characteristics were reported about the interviewer/facilitator? e.g. Bias, assumptions, reasons and interests in the research topic<br>*Participants were aware, and it is reported in the manuscript, that I am a Registered Nurse with an interest in how eczema knowledge is developed and shared between patients and clinicians in primary care and that this was a publically funded study.*	5
**Domain 2: study design**<br>*Theoretical framework*<br>9. Methodological orientation and theory<br>What methodological orientation was stated to underpin the study? e.g. grounded theory, discourse analysis, ethnography, phenomenology, content analysis<br>*In the methods section I explain that this is an exploratory qualitative study and that data analysis mainly involved constant comparison.*	5 and 6
Participant selection<br>10. Sampling How were participants selected? e.g. purposive, convenience, consecutive, snowball<br>*I selected participants using maximum variation purposive sampling to ensure a mix of ages, gender and ethnic background.*	5
11. Method of approach How were participants approached? e.g. face-to-face, telephone, mail, email<br>*Practitioners were recruited via the local Clinical Research Network and word of mouth and lay participants though a higher education institute website with mass sharing via word of mouth and media posts*	5
12. Sample size How many participants were in the study?<br>*32 interviews were completed*	5
13. Non-participation How many people refused to participate or dropped out? Reasons?<br>*No participants dropped out.*	NA
Setting<br>14. Setting of data collection Where was the data collected? e.g. home, clinic, workplace<br>*Interview data were collected in workplaces or on the telephone*	6

For peer review only

BMJ Open Eczema mindlines COREQ 27.11.18

16. Description of sample What are the important characteristics of the sample? e.g. demographic data, date<br>Interview participants were sampled by age, gender and ethnicity.	Tables 2 and 3<br>5
Data collection<br>17. Interview guide Were questions, prompts, guides provided by the authors? Was it pilot tested?<br>*A topic guide was used for interviews*	Table 1<br>5
18. Repeat interviews Were repeat interviews carried out? If yes, how many?<br>*No repeat interviews were carried out*	NA
19. Audio/visual recording Did the research use audio or visual recording to collect the data?<br>*Interviews were audio recorded*	5
20. Field notes Were field notes made during and/or after the interview or focus group?<br>*No*	NA
21. Duration What was the duration of the interviews or focus group?<br>*Interviews lasted from 16-55 minutes*	6
22. Data saturation Was data saturation discussed?<br>*Yes*	6
23. Transcripts returned Were transcripts returned to participants for comment and/or correction?<br>*No*	NA
**Domain 3: analysis and findings**<br>*Data analysis*<br>24. Number of data coders How many data coders coded the data?<br>*I analysed the data independently.*	6
25. Description of the coding tree Did authors provide a description of the coding tree?<br>*No*	NA
26. Derivation of themes Were themes identified in advance or derived from the data?<br>*Findings were derived inductively from the data*	7
27. Software What software, if applicable, was used to manage the data?<br>*No*	NA
28. Participant checking Did participants provide feedback on the findings?<br>*No*	NA
Reporting<br>29. Quotations presented Were participant quotations presented to illustrate the themes / findings? Was each quotation identified? e.g. participant number<br>*Quotations are provided and attributed to individuals.*	6-12
30. Data and findings consistent Was there consistency between the data presented and the findings?<br>*Yes.*	6-12
31. Clarity of major themes Were major themes clearly presented in the findings?<br>*Yes.*	6-12
32. Clarity of minor themes Is there a description of diverse cases or discussion of minor themes?<br>*Yes, I present a broad spectrum of participant's views.*	6-12

For peer review only

BMJ Open

Knowledge mobilisation: an exploratory qualitative interview study to confirm and envision modification of lay and practitioner eczema mindlines to improve consultation experiences and self-management in primary care in the United Kingdom

Journal:	*BMJ Open*
Manuscript ID	bmjopen-2018-028225.R1
Article Type:	Research
Date Submitted by the Author:	10-Apr-2019
Complete List of Authors:	Cowdell, Fiona; Birmingham City University, Faculty of Health Education and Life Sciences
**Primary Subject Heading**:	Dermatology
Secondary Subject Heading:	General practice / Family practice, Health services research, Nursing, Public health, Qualitative research
Keywords:	Mindlines, Knowledge mobilisation, Eczema < DERMATOLOGY, PRIMARY CARE, Self-management, Consultation experiences

SCHOLARONE™
Manuscripts

BMJ Open Mindline confirmation R1 10.4.19

Title Page

Article title: Knowledge mobilisation: an exploratory qualitative interview study to confirm and envision modification of lay and practitioner eczema mindlines to improve consultation experiences and self-management in primary care in the United Kingdom

Author information:
Fiona Cowdell
DProf, RN
Professor of Nursing and Health Research
Faculty of Health, Education and Life Sciences
Birmingham City University
220 Ravensbury House
Westbourne Road
Edgbaston
Birmingham
B15 3TN
UK
Telephone: +44 (0)121 300 4345
Email: Fiona.cowdell@bcu.ac.uk
orcid.org/0000-0002-9355-8059

Word count: 4771 (including tables)

Number of figures and tables: Figures 2, Tables 4

Key words: *Ba*, consultation, eczema, knowledge mobilisation, mindlines, primary care, self-management, qualitative

Disclaimer and source of support:
This article presents independent research funded by the National Institute for Health Research (NIHR). The views expressed are those of the author and not necessarily those of the NHS, the NIHR or the Department of Health.

For peer review only

BMJ Open Mindline confirmation R1 10.4.19

Knowledge mobilisation: an exploratory qualitative interview study to confirm and envision modification of lay and practitioner eczema mindlines to improve consultation experiences and self-management in primary care in the United Kingdom

Abstract

Objectives: To investigate whether initial eczema mindlines, "collectively reinforced, internalised, tacit guidelines", are an accurate representation of the experiences of lay people and practitioners in primary care and to explore how these mindlines may best be revised to improve eczema care.

Design: Exploratory qualitative interviews with constant comparative analysis and data mining.

Setting: United Kingdom, primary care

Participants: People with eczema or parents of children with eczema (n=19) and primary care practitioners (n=13).

Results: Interview data were analysed using constant comparison of new data with existing initial eczema mindlines to identify areas of agreement and disagreement. Data were mined for participant's thoughts about *whose* mindlines should be modified, *how* this may be achieved and *what* core content is essential. Eczema mindlines and the spiral of knowledge creation, from which they evolved, intuitively made sense. Participants offered examples of how their eczema knowledge is continually produced and transformed as they interact with others. They reported diverse and wide-ranging influences on their thinking and recognised the critical relationship between lay and practitioner mindlines. For this reason they advocated modifying lay and practitioner mindlines in parallel. Participants advised amendment based on consistent information directed to all who influence eczema care. Information should come from trusted sources and be easy to access, distilled, practical, contextually relevant and amenable to assimilation.

Conclusions: The purpose here is to improve primary care consultation experiences and self-management in eczema. The remaining challenge is to find novel, simple and pragmatic methods of modifying eczema mindlines to instill shared and consistent understanding. Given the prevalence of eczema and the scope of people who influence self-care, interventions should transcend patient-practitioner boundaries and address the wider community. One conceptually congruent approach is to create a *Ba*, which in this case would be a virtual space for generating and sharing eczema knowledge.

For peer review only - http://bmjopen.bmj.com/site/about/guidelines.xhtml

BMJ Open Mindline confirmation R1 10.4.19

Strengths and limitations

- Novel study confirming and extending existing lay and practitioner eczema mindlines
- Diverse sample of UK patients and parents of children with eczema and primary care practitioners
- Enhanced understanding of *whose* mindlines should be modified and *when* and *how* offers a starting point for the complex process of mindline amendment.
- Combining of parent and adult with eczema data and data from different practitioner groups may have limited recognition of nuanced differences in mindlines

For peer review only

For peer review only - http://bmjopen.bmj.com/site/about/guidelines.xhtml

Introduction

Eczema is a common and burdensome long-term skin condition (1) predominantly treated in primary care (2). People seek advice from general practitioners (GPs), nurses, health visitors and pharmacy staff. Consultations with GPs can be frustrating for both patient (3) and practitioner (4, 5) and there may be considerable differences between beliefs about assessment and treatment (6). Nurse consultations tend to be more positively regarded (7, 8). Research into the role of pharmacy staff is limited (9).

The essence of eczema treatment in primary care is the regular application of emollients, at least daily and often for many years, with or without intermittent use of topical corticosteroids (TCS). Non-adherence is common due to high self-management demand (10) and limited, sometimes conflicting, information (11). Effective self-management can improve disease outcomes and quality of life (12). Interventions to support eczema self-management are poorly understood, are not available to all, can be resource intensive and have varying impact (13). Eczema is not formally categorised as a long-term condition (14).

Knowledge mobilisation (KM) is concerned with the creation and movement of new knowledge to where it can be most useful (15). To be effective KM activity must be relational, constructed from social interaction and context-specific (16-18). One method of mobilising knowledge is amending mindlines. Mindlines were developed from the work of Polyani (19) and Nonaka & Takeuchi (20) specifically the concept of the **S**ocialization, **E**xternalization, **C**ombination, **I**nternalization (SECI) spiral in which knowledge is perpetually produced and transformed as users interact, collaborate and learn (17). Mindlines are "collectively reinforced, internalised tacit guidelines". They lie beneath clinical decision-making (21) and are underpinned by acceptance that tacit knowledge (knowledge that is implicit and hard to simply transfer to another person) is a more powerful influencer of action than formal codified knowledge (knowledge that can easily be articulated, accessed and shared through mainstream approaches such as documents, educational videos and the like) (19, 20). Mindlines are predicated on the notion of a flexible, embodied and intersubjective understanding of knowledge, the existence of multiple realties and acceptance of knowledge being context-specific (17). In their seminal work Gabbay and LeMay identified mindlines amongst practitioners in primary care (21); this and subsequent work has been frequently cited. However, minimal investigation has been undertaken on condition specific mindlines or the existence of the lay (patient) equivalent of mindlines (22).

For peer review only - http://bmjopen.bmj.com/site/about/guidelines.xhtml

A recent ethnographic study investigated the construction of healthcare practitioner and lay eczema mindlines in primary care (3, 23). From this, initial models of eczema mindlines were developed. For practitioners eczema was a low priority condition for which the "*recipe doesn't change".* Their mindlines were built on brief and limited early career education, general and focused internet searches, local formularies and guidance, tacit knowledge and interactions with patients and, to some extent, other practitioners. Differences in mindline development were noted between professions (23). On the whole they perceived limited need to update their eczema knowledge. The initial model of lay (person with eczema or caring for a child with the condition) mindlines indicated that, like practitioners, their knowledge was built up over time from multiple sources. They comprise interactions with family, friends and the wider community and with practitioners, general and focused internet searches, media coverage, trial and error and tacit knowledge. The desire for knowledge was most marked when people were self-managing by default, those who had become disillusioned and had disengaged from primary care (3).

The purpose of the study reported here is to: (i) investigate whether the initial practitioner and lay mindlines are an accurate representation of the realities experienced by practitioners and lay people in primary care and (ii) consider how eczema mindlines may best be revised or modified by adding reliable and useful knowledge and by erasing outdated or inaccurate information thus potentially improve quality of eczema consultations and self-management.

Methods

Aims

To: (i) investigate whether initial eczema mindlines, developed from previous research by the author of this paper (3, 23), are an accurate representation of the realities experienced by practitioners and lay people in primary care and (ii) consider how they may best be revised or modified.

Design

Exploratory qualitative interviews (24) with constant comparative data analysis (25).

Setting, participants and process

Data were collected by the author, a nurse and researcher, from December 2017-May 2018. Practitioners were recruited via the local Clinical Research Network and word of mouth and lay participants though a higher education institute website with mass sharing via word of mouth and media posts in an effort to recruit hard-to-reach groups who may not realise their value in this type

For peer review only - http://bmjopen.bmj.com/site/about/guidelines.xhtml

of study (26). Maximum variation purposive sampling was applied (27); characteristics were broad and focused on participant's ability and willingness to discuss eczema mindlines by virtue of their knowledge and experience. Single, individual, semi-structured, audio-taped interviews using a topic guide (table 1) were conducted. The topic guide was based on previous research by the author (3, 23) and questions likely to elicit thoughtful and detailed responses to address the research questions. Participants comprised lay people (n=19, table 2) and practitioners with between one and 26 years clinical experience (n=13, table 3) and lasted from 16 – 55 minutes. Interviews continued to the point of data sufficiency; that is until the depth and detail in the data gathered fully addressed the research questions (28). Most interviews were face-to-face, in the participant's workplace, and a small number via telephone. In preparation, participants were sent a brief explanation of mindlines and illustrations of the relevant initial eczema mindline (3, 23).

At the beginning of each interview the concept of mindlines and illustrations were discussed.<br>• How does this eczema mindline make sense to you?<br>• What are the similarities and differences in the way in which you know and think about eczema?<br>• How could eczema mindlines best be amended?<br>• Do you think there particular information that needs to be added or erased from existing mindlines?

Table 1: Interview topic guide

For peer review only - http://bmjopen.bmj.com/site/about/guidelines.xhtml

BMJ Open Mindline confirmation R1 10.4.19

Self / child	Age	Gender
Self	28	Female
Self	36	Male
Child	6	Female
Self	53	Male
Self	34	Female
Children	13 and 15	Female
Self	75	Female
Self	26	Female
Self	49	Male
Self	28	Female
Self	39	Female
Child	5	Female
Self	24	Male
Child	7	Female
Children	17 and 20	Female
Self	22	Female
Self	56	Male
Self	38	Male
Self	48	Female

Table 2: Demographic details of lay interview participants

Role	Gender
Dermatology nurse in primary care	Female
Dermatology nurse in primary care	Female
Community pharmacist	Female
Trainee general practitioner	Male
Practice nurse	Female
Nurse practitioner	Male
General practitioner	Male
General practitioner	Male
General practitioner	Female
General practitioner	Male
General practitioner	Male
Practice nurse	Female
Practice nurse	Female

Table 3: Demographic details of practitioner interview participants

Data analysis

Data collection and analysis were iterative processes (29, 30). Audio-data was professionally transcribed and transcripts proof read against recordings for accuracy. Data were analysed in two ways. Firstly in a constant comparison approach (25) incoming data was compared with data from the two previous studies from which initial eczema mindlines had been developed (3, 23). Here the focus was on identifying similarities and differences in the ways that mindlines are generated,

For peer review only - http://bmjopen.bmj.com/site/about/guidelines.xhtml

embedded and transformed over time. Secondly, through subsequent readings of the transcripts, data were mined for participant's thoughts about how mindlines may best be amended and their beliefs about specific information that should be added or erased.

Reflexivity

A reflexive attitude was maintained throughout, acknowledging own subjectivity and positioning as a nurse and skin health researcher and the influence that this may have on the study (31).

Ethical issues

The study was approved by a University Ethics Committee and the Health Research Authority. Written consent was taken for interviews and participants consented to publication of anonymised quotes.

Patient and public involvement

Lay people were involved in developing the research question and designing the study. Results will be communicated to participants in a brief summary.

Results

The results are presented in four sections. Firstly the extent to which lay and then practitioner eczema mindlines were perceived as being an accurate representation of their realities is considered. Areas of individual similarity and difference are considered. Secondly areas of connection are identified, followed by examples of synergy and dissonance between lay and practitioner mindlines. Finally a synthesis of *whose* mindlines should be amended with *what* information and *how* this could best be achieved is offered.

1a) Are lay eczema mindlines a reasonable representation of realities?

Lay participants reported that the concept of mindlines intuitively made sense and noted broad congruence in sources of knowledge and beliefs. They reviewed each element of the existing lay eczema mindline, that is: interactions with family, friends and the wider community and with practitioners, general and focused internet searches, media coverage, trial and error and tacit knowledge within the context of being content to self-manage or accept practitioner management or self-managing by default. Lay participants spoke at length about the presented mindline and provided examples and nuanced insights into the how, why and when their mindlines had been constructed.

For peer review only - http://bmjopen.bmj.com/site/about/guidelines.xhtml

Interactions with family and friends and the wider community were experienced by all. The most influential information was advice from mothers. Others reported picking up "*snippets*" of information, sometimes unbidden "*I've had someone on the Tube recommending a moisturiser*" (Female, 22). Suggestions could be overwhelming *"they were telling me their regimes or oh try this cream. Everybody had a cream to recommend and it got to the point where I was using so many things, you didn't know what was working from what wasn't"* (Female, 27) and often unfounded *"hearsay ... 'oh well so and so tried this', 'oh I know someone who did this'"* (Female, 28). Advice from practitioners was sometimes externally validated with other lay people before making a decision about treatment use.

Participants mainly spoke about GP consultations which were frequently viewed as unsatisfactory. GPs were perceived as having limited expertise "*sometimes I get the impression that they're doing no more than you would by Googling it"* (Female, 34) and working on a trial and error basis "*it's generally a bit of chuck everything at the wall and see what sticks"* (Male, 36). In contrast to the initial mindline the pathway of knowledge between lay people and practitioners was perceived as one-way. People experienced their views and experiences as being undervalued and suggested that they were provided with standard, often unhelpful, information, "*I think doctors think everyone's stupid …… I know the advice is well meant but it's generalistic"* (Male, 56). Some mentioned power differentials as the cause of their expertise being dismissed "*I interact with them as equal but he couldn't have that"* (Male, 53).

Lay participants also consulted with nurses, pharmacy staff, health visitors and a physician's assistant. These meetings were more positively evaluated, for example after seeing a nurse "*I felt much more confident that they know in detail what they were dealing with …… they seemed to have a lot more knowledge about products"* (Female, 27). Pharmacists were another source of practical and personalised advice "*I saw the pharmacist at the practice she was actually very, very helpful, she came out with this keep the creams in the fridge, stroke them on ……… the two saviours were the pharmacists really"* (Female, 75).

Most lay participants had searched the internet, the focus was invariably on finding new treatments or cure rather than understanding the condition. Most had used trusted sources such as NHS Choices, but for some this was "*a lovely set of generic blandishments"* (Male, 36). Many had "Googled" but were selective in using this information, "*lot of self-censoring, you know a filter*"

For peer review only - http://bmjopen.bmj.com/site/about/guidelines.xhtml

(Female, 34). Finding reliable information electronically was challenging and some withdrew *"I'm wary of Google in case you get frightened by things that you don't need to see"* (Female, 34).

Other media sources, including leaflets, newspapers and magazines, television programmes and social media, were used to a lesser degree and also treated judiciously. Few people read patient information leaflets enclosed with medications describing them as of little practical value. Leaflets in GP surgeries and pharmacies were infrequently taken and poorly evaluated. A few read newspapers and magazines but were sceptical "*I'm quite an avid newsreader, periodically there are articles … don't always believe 100% because I think there could be something sponsored"* (Female, 39). Participants reported seeing occasional television programmes that influenced their thinking but not often their actions; the BBC was considered most trustworthy. Participant's valued social media for example Facebook and Twitter, the value here was perceived reality, "*a fellow sufferer saying 'this works for me*'" [Female, 34]. As with other knowledge sources participants filtered information, "*the critical eye that I have definitely makes me check and double check, are these valid sources and cross referenced?"* (Female, 26).

Trial and error was a familiar and frustrating experience for every lay participant "*you build up this medicine cabinet full of every ointment and then you have a set of stories about each one"* (Male, 53). Tacit knowledge was viewed as an amalgamation of personal experience accrued over time and hearsay from unknown or unremembered sources often from the wider community. Universal tacit knowledge was that TCS "*thin the skin*" and should be avoided as far as possible. No participants could trace how this thought had entered their mindline, "*I just grew up with that 'oh you have to be careful with this'"* (Female, children 13&15) and "*umm … I'm trying to think who told me*" (Female 34).

Participants identified with being content to self-manage or accept practitioner management or self-managing by default. Most described moving back and forth between these states, for example, at times of eczema flare, treatment failure or unsatisfactory consultations. These circumstances triggered fresh attempts to find new ways of managing their eczema sometimes with clouded judgment, *"we just jump to unreliable sources or do irrational things because you can be tempted to, when you've got a really bad flare up"* (Female, 26).

Eczema mindlines instinctively made sense to lay participants and the content presented resonated with their thoughts, beliefs and experiences. They recognised the multiple influences, including the power of social interactions. Their analysis of personal eczema mindlines reinforced the complexity

For peer review only - http://bmjopen.bmj.com/site/about/guidelines.xhtml

and difficulty in unravelling how all the elements coalesce particularly as, although content is similar for each individual, the way in which mindlines have developed over time had subtle variations. Lay participants linked efforts to amend mindlines closely with different phases of self-management and for this reason suggested that this should be integrated into the mindlines image (see figure 1).

1b) Are practitioner eczema mindlines a reasonable representation of realities?

Practitioners agreed with the initial mindline but offered less discussion, for example "*so I think it's nice model actually, I think it's all fair, everything you've said is fair and reasonable and appropriate*" (Male GP). All agreed that eczema is not perceived as a priority in primary care, that treatment is limited by their local emollient formulary and that they exercised caution in prescribing TCS. A dominant influence on the mindlines of the four practitioners with self-reported expertise in eczema care was exposure to dermatology experts in secondary care. The trusted and structured guidance given had become firmly embedded leading to increased knowledge, skills and confidence and "*braver*" prescribing of non-formulary emollients (when clinically appropriate) and TCS. These experts contested the backdrop of low priority but concurred with the relative straightforwardness of treatment regimens whilst viewing these not as a "recipe" but as principles that should be individualised. The practitioner mindline illustration was amended for clarity (figure 2).

2) Areas of lay and practitioner mindline connection

Unprompted participants considered mindlines of the "other" and the interplay between these entities. Lay participants suggested a need for change in practitioner mindlines but focused on amending lay mindlines. Practitioners were certain about the best targets for mindline amendment. GPs stated that their own and lay mindlines should be amended but considered the former unlikely due to the low priority given to eczema. This view was reinforced by an expert nurse, "*GPs are entrenched in their views*" and a lay participant, "*it's a challenge with the entrenched stubbornness of the medical profession, people will not change their beliefs once they 'know something'*" (Lay male, 36). Other practitioners advised that health visitors, nurses and community pharmacy staff should be targeted given their relative accessibility for patients.

A challenge for all participants was the volume of often contradictory information from a multitude of sources available to lay people. A GP recounted trying to persuade a patient to use TCS properly, "*I validate it with something and say, look I do extra work in dermatology, please just trust me, this is fine*". However his efforts were thwarted by the patient's existing mindlines and standard advice given by pharmacists which generated fear.

For peer review only - http://bmjopen.bmj.com/site/about/guidelines.xhtml

Practitioners almost universally identified the key reliable and useful knowledge that should be added to, and outdated or inaccurate information that should be erased from, existing mindlines. Core content is summarised in quotations from participants (table 4). Lay participants were less certain about the essential content but agreed key issues included understanding and accepting that eczema may be a long-term condition requiring ongoing treatment with emollients and that TCS have a place in the treatment of flares.

- *"never let anyone say to you again that eczema is just a bit of dry skin"*
- *"treat as a long term condition"*
- *"eczema needs maintenance treatment"*
- *"mantra of the best emollient is the one that [is used]"*
- *"don't be frightened [of] steroids and use them properly"*
- *"when it does flare up you've got to hit it hard quickly with your steroid"*

Table 4: Core eczema mindline content from practitioners

3) Examples of synergy and dissonance between lay and practitioner mindlines

Returning to the original definition of mindlines as "collectively reinforced, internalised tacit guidelines" participants demonstrated both the complexity in development and the influential interplay between lay and practitioner mindlines. Participants identified areas of synergy and dissonance across and between mindlines. A key finding is that eczema knowledge does not involve just people living with the condition and practitioners but rather there is a pervasive social element, for example the received wisdom or "*old wives tale*" that TCSs "*thin the skin*" causing irreparable damage.

Perceived need to update was low among most practitioners and this pervaded some lay mindlines "*there's no point in wasting the doctors time*" (Male, 53). As a result eczema was sometimes relegated to an additional concern rather than the primary reason for consultation and then necessarily given limited attention by practitioners, thus reinforcing its low priority. Practitioners reported *"we don't often think about it as a chronic condition which needs regular follow up and a defined plan"* (Male, trainee GP), leading to reactive treatment with few follow-up appointments. Lack of maintenance therapy impacted on self-management leading to a cycle of flare management rather than a long-term, agreed management plan.

For peer review only - http://bmjopen.bmj.com/site/about/guidelines.xhtml

BMJ Open Mindline confirmation R1 10.4.19

4) Mindline amendment: whose, what and how?

Mindline amendment proved a conundrum to all. Lay participants reflected on the futility of simply providing more information. Immediate suggestions including predictable approaches such as websites media and leaflets were made but rapidly dismissed. For example, *"website … but who would go on it?* (Male, 53) and "*leaflets in GP surgeries, but then you just get information fatigue*" (Male, 49). Similarly suggestions about using social media and 'apps' were voiced and rejected within the same sentence. Interactive fora were vetoed due to the need for leadership and moderation. One participant favoured a "*Facebook group specifically for eczema care, I'd probably be part of that ... good to have a safe place for people to talk*" (Female, 22) although she acknowledged that sustained participation was unlikely.

Pivotal factors in lay mindline amendment included "realness", exemplified by "*that's the stuff that I'm interested in and that I'll home in on are the forums where you've got people telling their stories*" (Male, 53) and "*people work off stories don't they, like, I'm living proof*" (Male, 36). Other people's stories were persuasive for some and disliked by others. Trustworthiness, ease of access and applicability to self were important, "*quick trusted and easy to access*" (Female, 34) from trusted sources "*pharmacies, a credible source of information*" (Female, children 17&20). All lay participants disliked the generic nature of current information "*eczema is very particular, people regard it as part of who they are, because it does shape what you're doing*" (Male, 53). Disillusionment and lack of motivation to change was identified as a challenge to mindline amendment. Interactions and relationships were addressed on several levels. Engagement was one hurdle "*you need a way of hooking people in, people who are likely to walk past and go 'I haven't got time to care about that'*" (Female, 34). The impact of social and relational factors suggested a need for wider amendment "*an effective way would be to normalise this thing, there's still a shame element around it, bringing it into normal conversations, everyday discussions, so it's not a thing that you're just hiding away*" (Female, 26). Shared, consistent information could be powerful in eradicating influential "*old wives tales*".

Practitioners proposed existing educational mechanisms for amending mindlines including: free taught sessions with "*really, really practical, emollient testing*" (Female, nurse) and information which can readily be applied in everyday practice; free online education; sessions during protected learning time. Practitioners rarely accessed available eczema education and were "*wary of messages from pharma or perceived messages from pharma*" (Female, GP). Expert practitioners were

For peer review only - http://bmjopen.bmj.com/site/about/guidelines.xhtml

exasperated by the lack of motivation for change amongst generalist colleagues and the continued provision "*outdated and wrong*" advice (Female, nurse). Practitioners were aware of existing patient-focused resources, for example NHS Choices, patient.co.uk, the Primary Care Dermatology Society and the British Association of Dermatologists Patient Information Leaflets, but rarely signposted or used these in consultations.

Participants pointed to the need for shared understandings and consistent advice through synergistic mindlines. Reflection on mindlines brought to the fore the diversity and magnitude of influencing factors and the extent to which knowledge was accessed in different circumstances, for example during an eczema flare. The need for consistency of information was a high priority and one best addressed by seeking to modify the multiple channels of information that contribute to lay and practitioner eczema mindlines in primary care with straightforward, evidence based messages.

Discussion

The first aim of this study was to investigate whether initial eczema mindlines are an accurate representation of the experiences of practitioners and lay people in primary care. Mindlines intuitively made sense to, and were confirmed by, all participants. Areas of synergy and dissonance were identified, as was the interplay between lay and practitioner eczema mindlines. Secondly mindline amendment was investigated. The need to modify and unify mindlines by adding reliable and useful knowledge and by erasing outdated or inaccurate information was universally agreed. However, strategies to achieve amendment proved elusive. The prevalence of eczema, the diversity of *how* and *when* mindlines are developed suggests a need to find focused strategies for wide-reaching, societal amendment to ensure simple, consistent messages to improve consultation experiences and self-management in primary care. Practitioners offered suggestions about essential areas for amendment.

This study is one of few to apply mindline theory to a specific condition and is original in identifying the relationship between lay and practitioner mindlines. The study conforms to the conventions of qualitative research in being rigorous (externally auditable thorough clear reporting), relevant (enhancing understanding of the subject), resonant with reader's experiences and understandings and reflexive (31). The study has deepened understanding of *whose* mindlines should be modified and *when* and *how* this may best be achieved. This offers a robust starting point for the complex amendment process. Limitations of the study include parent and adult with eczema data being combined, potentially limiting nuanced differences in mindlines. Similarly practitioner mindlines have been viewed as a whole. That said, mindlines are individual and can change over time.

For peer review only - http://bmjopen.bmj.com/site/about/guidelines.xhtml

To date little attention has been given to the construction of lay (or patient) mindlines. Gabbay and LeMay (17) suggest a patient equivalent, but this possibility is poorly represented in other literature (22). The term clientlines appears in one study (32) but is not fully explored. However, the notion that knowledge creation inevitably happens in patients is not contested (33) and is extended here in the breadth of influences on lay eczema mindlines. This could be attributable to prevalence, with most people knowing someone with eczema and the perception that it is a "health problem which is not an illness" (34) and therefore perceived as open to treatment suggestions from anyone.

Amendment of mindlines is rarely reported, a synthesis of 10 years of literature (n=340) identified 28 related to practitioner amendment. These emphasise the importance of collaborative learning, relationship building and effective leadership in the development of valid, collective, evidence-based mindlines (22). Repeating the search strategy used for this review in August 2018 revealed an abundance of new literature (n=422) but still little directly addressing how mindlines may best be amended. Allied literature indicates the need for a judicious approach to amending practitioner mindlines. Primary care practitioners are necessarily expert generalists (35) and have to know about many conditions. It is clearly impossible for any individual to process all available information. Practitioners use coping strategies such as "satisficing" that is, curtailing the amount of information gathered to enable them to make a "good enough" decision (36). Given the view that eczema is straightforward with treatment being standard and unchanging over the years, motivation to change practice is limited (23). Likewise treatment decisions are apparently made rapidly, perhaps fitting somewhere between fast, automatic, System 1 thinking and the more deliberative, conscious slow and effortful System 2 approach (37) and instead accessing knowledge from a well-worn path. The danger here is that *this* path may not lead to the best outcomes.

Mindlines evolved from the work of Polyani and Nonaka & Takeuchi, in particular, the SECI spiral (17) in which knowledge is perpetually produced and transformed as users interact, collaborate and learn (38). Gabbay and LeMay (21) point to the critical nature of knowledge-in-practice-in-context, in which in each context new knowledge is converted by complex social processes. This current study concurs, finding that eczema knowledge is founded in a wider community (39, 40), sometimes facilitated by others (41) and often influenced by numerous, interacting personal and social factors (42). To date moves to improve eczema self-management and consultation experiences largely comprise educational and psychological interventions (13, 43). This study offers an alternative socially-mediated approach. Returning to the work of Nonaka offers new insights into mindline

For peer review only - http://bmjopen.bmj.com/site/about/guidelines.xhtml

amendment drawing on the relationship between the SECI spiral and *Ba*. *Ba*, originally discussed by the Japanese philosopher Nishida (44, 45) and later refined by Shimizu (46), is a shared space for knowledge generation and sharing. *Ba* is not about the space per se (47), rather it is a shared context-in-motion which advances over time and has no fixed boundaries or membership. It is about the here-and-now and thrives on people with different viewpoints (48). *Ba* space occurs naturally, is not prescribed, relationships are fluid, permeable and iterative (49, 50). It has four characteristics: originating (face-to-face, individual communications), interacting (face-to-face group dialogue), cyber (virtual group interactions) and exercising (virtual individual interactions) (50). In the main *Ba* has been considered in business organisations (51-56). More recently attention has been given to its application in other contexts, for example in making "big data" widely useful (57) and in supporting educational endeavours (50, 58, 59). To date there is no evidence of attempting to create *Ba* across lay-practitioner-community boundaries. Given the need to amend mindlines, both lay and practitioner, across the community the notion of deliberatively creating *Ba* and integrating innovative approaches to addressing each of the four characteristics in a specific geographic area offers a new opportunity for eczema knowledge mobilisation. This work is currently underway and will be the focus of future publications.

In some respects core treatment of eczema in primary care is relatively straightforward. Experts in the field propose "getting control" and "keeping control" (60) which requires emollient maintenance to reduce incidence and severity of flares and need for sufficient use of the right potency TCS when required. The key to effective self-management is supporting people to do these two things well and addressing the enormous challenges they face on a day-to-day enduring basis (61-64).

This study offers a new approach to improving consultation experiences and self-management through active and community-wide mindline amendment to develop consistent and shared understandings. Core reliable and useful knowledge that needs to be integrated, and outdated and inaccurate knowledge to be erased is identified. Mechanisms for change need to be socially-mediated, multi-faceted, tailored to specific groups and convey consistent, evidence based, core messages.

For peer review only - http://bmjopen.bmj.com/site/about/guidelines.xhtml

BMJ Open Mindline confirmation R1 10.4.19

Contribution statement

FC is the sole contributor to this paper

Funding

This article presents independent research funded by the National Institute for Health Research (NIHR). The views expressed are those of the author and not necessarily those of the NHS, the NIHR or the Department of Health.

Ethical approval

The study was approved by a University Ethics Committee. Written consent was taken for interviews. Participants consented to publication of anonymised information.

Competing interests

None to declare

Acknowledgements

Thanks to Jay Nolan-Latchford for the mindline illustrations and to all the participants who shared their time and thoughts.

Data sharing statement

The datasets generated and/or analysed during the current study are not publicly available as they are not designed to be re-analysed by others but are available from the corresponding author on reasonable request.

For peer review only

For peer review only - http://bmjopen.bmj.com/site/about/guidelines.xhtml

References

1. Hay RJ, Johns NE, Williams HC, et al. The global burden of skin disease in 2010: an analysis of the prevalence and impact of skin conditions. J Invest Dermatol. 2014; 134: 1527-1534.
2. Schofield J, Grindlay D, Williams H, Skin conditions in the UK: a health care needs assessment. Centre of Evidence Based Dermatology, University of Nottingham; 2009.
3. Cowdell F. Knowledge mobilisation: an ethnographic study of the influence of lay mindlines on eczema self-management in primary care in the United Kingdom. BMJ Open. 2018;8:e021498.
4. Magin P, Adams J, Heading G, Pond C. Patients with skin disease and their relationships with their doctors: a qualitative study of patients with acne, psoriasis and eczema. Med J Aust. 2009; 190:62-64
5. Santer M, Burgess H, Yardley L, et al. Experiences of carers managing childhood eczema and their views on its treatment: A qualitative study. Br J Gen Pract. 2012; 62:261-267.
6. Powell K, Le Roux E, Banks J, et al. GP and parent dissonance about the assessment and treatment of childhood eczema in primary care: a qualitative study. BMJ Open. 2018; 8:e019633.
7. Nicol NH, Ersser SJ. The role of the nurse educator in managing atopic dermatitis. Immunol Allergy Clin. 2010; 30:369-383.
8. Rolinck-Werninghaus C, Trentmann M, Reich A, et al. Improved management of childhood atopic dermatitis after individually tailored nurse consultations: a pilot study. Pediat Allergy Immunol. 2015; 26: 805-810.
9. Tucker R, Stewart D. Why people seek advice from community pharmacies about skin problems. Int J Pharm Pract. 2015; 23(2): 150-153.
10. Santer M, Burgess H, Yardley L, et al. Managing childhood eczema: Qualitative study exploring carers' experiences of barriers and facilitators to treatment adherence. J Adv Nurs. *2013*; 69:2493-2501.
11. Teasdale EJ, Muller I, Santer M. 2017. Carers' views of topical corticosteroid use in childhood eczema: a qualitative study of online discussion forums. Br J Dermatol. 2017; 176(6):1500-1507.
12. Department of Health 2014 The Mandate: A mandate from the Government to NHS England: April 2014 – March 2015 https://www.gov.uk/government/uploads/system/uploads/attachment_data/file/383495/2902896_DoH_Mandate_Accessible_v0.2.pdf Accessed 4.7.18
13. Ridd MJ, King AJL, Le Roux E, et al. Systematic review of self-management interventions for people with eczema. Br J Dermatol. 2017; 177(3):719-734.
14. Hanlon P, Daines L, Campbell C. et al. Telehealth interventions to support self-management of long-term conditions: a systematic metareview of diabetes, heart failure, asthma, chronic obstructive pulmonary disease, and cancer. JMIR. 2017; 19:e172.
15. Ward V. 2017. Why, whose, what and how? A framework for knowledge mobilisers. Evid Policy. 2017; 13:477-497.
16. Ferlie E, Crilly T, Jashapara A, et al. Knowledge mobilisation in healthcare: a critical review of health sector and generic management literature. Soc Sci Med. 2012;74:1297-1304.
17. Gabbay J, Le May A. Practice-based evidence for healthcare: clinical mindlines. London: Routledge; 2010.
18. Plamondon K, Caxaj S. 2018. Toward Relational Practices for Enabling Knowledge-to-action in Health Systems: The Example of Deliberative Dialogue. Adv Nurs Sci. 2018; 41:18-29.
19. Polanyi M. The logic of tacit inference. Philos.1966; 41:1-18.
20. Nonaka I, Takeuchi H. The knowledge-creating company: How Japanese companies create the dynamics of innovation. Oxford: Oxford University Press; 1995.

For peer review only - http://bmjopen.bmj.com/site/about/guidelines.xhtml

BMJ Open Mindline confirmation R1 10.4.19

21. Gabbay J, le May A. Evidence based guidelines or collectively constructed "mindlines?" Ethnographic study of knowledge management in primary care. BMJ. 2004; 329:1013.
22. Wieringa S, Greenhalgh T. 10 years of mindlines: a systematic review and commentary. Imp Sci. 2015; 10:45.
23. Cowdell F Knowledge mobilisation: An ethnographic study of the influence of practitioner mindlines on atopic eczema self-management in primary care in the United Kingdom *awaiting editor decision with BMJ Open*
24. Stebbins RA. Exploratory research in the social sciences. Sage; 2001 May 14.
25. Cho JY, Lee EH. Reducing confusion about grounded theory and qualitative content analysis: Similarities and differences. The qualitative report. 2014;19(32):1-20.
26. Bonevski B, Randell M, Paul C, Chapman K, Twyman L, Bryant J, Brozek I, Hughes C. Reaching the hard-to-reach: a systematic review of strategies for improving health and medical research with socially disadvantaged groups. BMC Med Res Meth. 2014 Dec;14(1):42.
27. Tongco MD. Purposive sampling as a tool for informant selection. Ethnobotany Research and Applications. 2007. 31; 5:147-58.
28. O'Reilly M, Parker N. 'Unsatisfactory Saturation': a critical exploration of the notion of saturated sample sizes in qualitative research. Qual Res. 2013; 13:190-197.
29. Spradley JP. Participant observation. Illinois: Waveland Press; 2016.
30. Spradley JP. The ethnographic interview. Illinois: Waveland Press; 2016.
31. Finlay L. Phenomenology for psychotherapists: Researching the lived world. Oxford: Wiley Blackwell; 2011.
32. Chandler CI, Jones C, Boniface G et al. Guidelines and mindlines: why do clinical staff over-dignose malaria in Tanzania? A qualitative study. Malar J 2008;7:53
33. Launer J. Guidelines and mindlines. Postgraduate medical journal. 2015 Nov 1;91(1081):663-4.
34. Cornwell J. Hard-earned lives: accounts of health and illness from East London: Routledge Kegan & Paul;1984
35. Reeve J, Dowrick CF, Freeman GK, et al. Examining the practice of generalist expertise: a qualitative study identifying constraints and solutions. JRSM short reports. 2013; 4(12):2042533313510155.
36. Bate L, Hutchinson A, Underhill J, et al. How clinical decisions are made. Br J Clin Pharmacol. 2012; 74:614-20.
37. Kahneman D. Thinking, Fast and Slow. London: Penguin; 2011.
38. Nonaka I, Nishihara AH, Kawada H. Knowledge-based management theory. In Knowledge Creation in Public Administrations 2018 (pp. 1-21). Palgrave Macmillan, Cham.
39. Dobele A, Fry J, Rundle-Thiele S, Fry T. Caring for baby: what sources of information do mothers use and trust? Journal of Services Marketing. 2017 Oct 9;31(7):677-89.
40. Reifegerste D, Bachl M, Baumann E. Surrogate health information seeking in Europe: Influence of source type and social network variables. International journal of medical informatics. 2017 Jul 1;103:7-14.
41. Edwards M, Wood F, Davies M, Edwards A. 'Distributed health literacy': longitudinal qualitative analysis of the roles of health literacy mediators and social networks of people living with a long-term health condition. Health Expectations. 2015 Oct;18(5):1180-93.
42. Bigsby E, Hovick SR. Understanding associations between information seeking and scanning and health risk behaviors: An early test of the structural influence model. Health communication. 2018 Mar 4;33(3):315-25.
43. Ersser SJ, Cowdell F, Latter S, Gardiner E, Flohr C, Thompson AR, Jackson K, Farasat H, Ware F, Drury A. Psychological and educational interventions for atopic eczema in children. Cochrane Database of Systematic Reviews 2014, Issue 1. Art. No.: CD004054. DOI: 10.1002/14651858.CD004054.pub3.
44. Nishida K, Dilworth DA. Fundamental Problems of Philosophy the World of Action and the Dialectical World. Translated with an Introd. By David A. Dilworth.

For peer review only - http://bmjopen.bmj.com/site/about/guidelines.xhtml

45. Nishida K. An Inquiry into the Good, as translated by Abe, M. and C. Ives.(1990). New Haven./London: Yale University. 1921.
46. Shimizu H. Ba-principle: new logic for the real-time emergence of information. Holonics. 1995 Aug;5(1):67-79.
47. Bartolacci C, Cristalli C, Isidori D, Niccolini F. Ba virtual and inter-organizational evolution: a case study from a EU research project. Journal of Knowledge Management. 2016 Jul 11;20(4):793-811.
48. Nonaka I, Toyama R. The knowledge-creating theory revisited: knowledge creation as a synthesizing process. Knowledge management research & practice. 2003 Jul 1;1(1):2-10.
49. Nonaka I, Konno N. Methodology of knowledge creation. Tokyo: Toyo Keizai. 2003 Apr 17.
50. Krahe JA, Lalley C, Solomons NM. Beyond Survival: Fostering Growth and Innovation in Doctoral Study–A Concept Analysis of the Ba Space. International journal of nursing education scholarship. 2014 Jan 1;11(1):11-8.
51. Nonaka I, Von Krogh G, Voelpel S. Organizational knowledge creation theory: Evolutionary paths and future advances. Organization studies. 2006 Aug;27(8):1179-208.
52. Von Krogh G, Nonaka I, Rechsteiner L. Leadership in organizational knowledge creation: A review and framework. Journal of Management Studies. 2012 Jan 1;49(1):240-77.
53. Fayard PM. Strategic communities for knowledge creation: A Western proposal for the Japanese concept of Ba. Journal of Knowledge Management. 2003 Dec 1;7(5):25-31.
54. Corno F, Reinmoeller P, Nonaka I. Knowledge creation within industrial systems. Journal of management and governance. 1999 Dec 1;3(4):379-94.
55. Hussi T. Reconfiguring knowledge management–combining intellectual capital, intangible assets and knowledge creation. Journal of knowledge Management. 2004 Apr 1;8(2):36-52.
56. Tyagi S, Cai X, Yang K, Chambers T. Lean tools and methods to support efficient knowledge creation. International Journal of Information Management. 2015 Apr 1;35(2):204-14.
57. Philip J. An application of the dynamic knowledge creation model in big data. Technology in Society. 2018 Apr 5.
58. de Aguiar TR, Paterson AS. Sustainability on campus: knowledge creation through social and environmental reporting. Studies in Higher Education. 2017 Feb 22:1-3.
59. Gupta V, Jain N. Harnessing information and communication technologies for effective knowledge creation: Shaping the future of education. Journal of Enterprise Information Management. 2017 Sep 11;30(5):831-55.
60. Williams HC. Preventing eczema flares with topical corticosteroids or tacrolimus: which is best?. British Journal of Dermatology. 2011 Feb;164(2):231-3.
61. Santer M, Burgess H, Yardley L, et al., Managing childhood eczema: Qualitative study exploring carers' experiences of barriers and facilitators to treatment adherence. J Adv Nurs. 2013; 69:2493-2501.
62. Jackson K, Ersser SJ, Dennis H, et al., The eczema education programme: Intervention development and model feasibility. J Eur Acad Dermatol Venereol. 2014; 28:949-956.
63. Santer M, Muller I, Yardley L, et al. 'You don't know which bits to believe': Qualitative study exploring carers' experiences of seeking information on the internet about childhood eczema. BMJ Open. 2015; 5:e006339-e006339. doi:10.1136/bmjopen-2014-006339
64. Santer M, Muller I, Yardley L, et al. Parents' and carers' views about emollients for childhood eczema: Qualitative interview study. BMJ Open. *2016; 6:* e011887-e011887. doi:10.1136/bmjopen-2016-011887

For peer review only - http://bmjopen.bmj.com/site/about/guidelines.xhtml

BMJ Open Mindline confirmation R1 10.4.19

Table 1: Interview topic guide

Table 2: Demographic details of lay interview participants

Table 3: Demographic details of practitioner interview participants

Table 4: Core eczema mindline content from practitioners

Figure 1: Lay eczema mindline

Revised lay eczema mindline

Figure 2: Practitioner eczema mindline

Revised practitioner eczema mindline

For peer review only

For peer review only - http://bmjopen.bmj.com/site/about/guidelines.xhtml

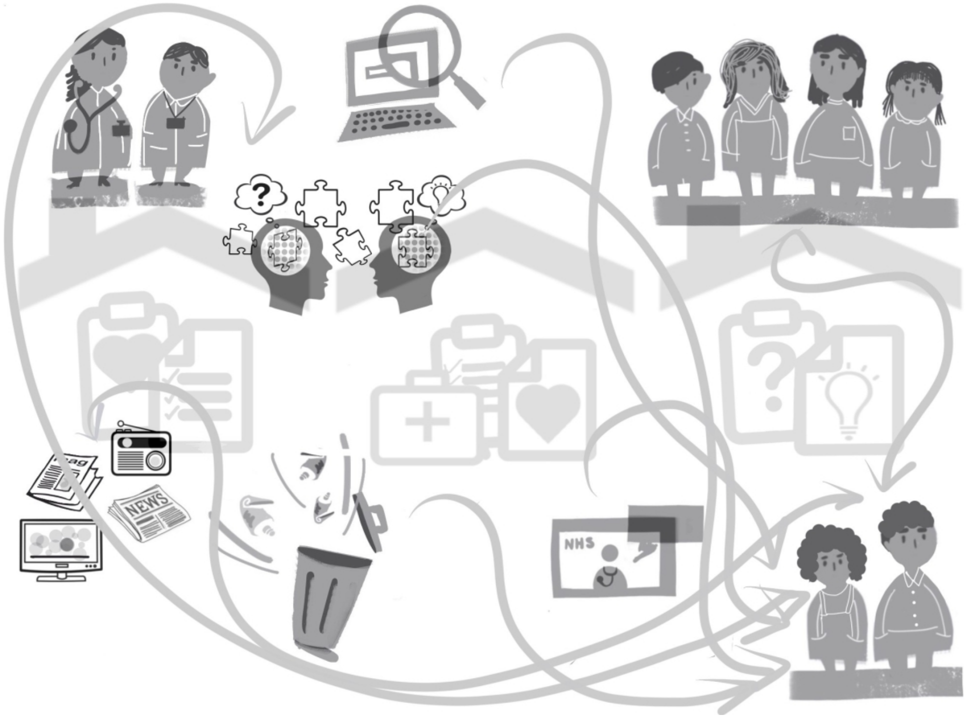

Lay eczema mindline

394x295mm (132 x 132 DPI)

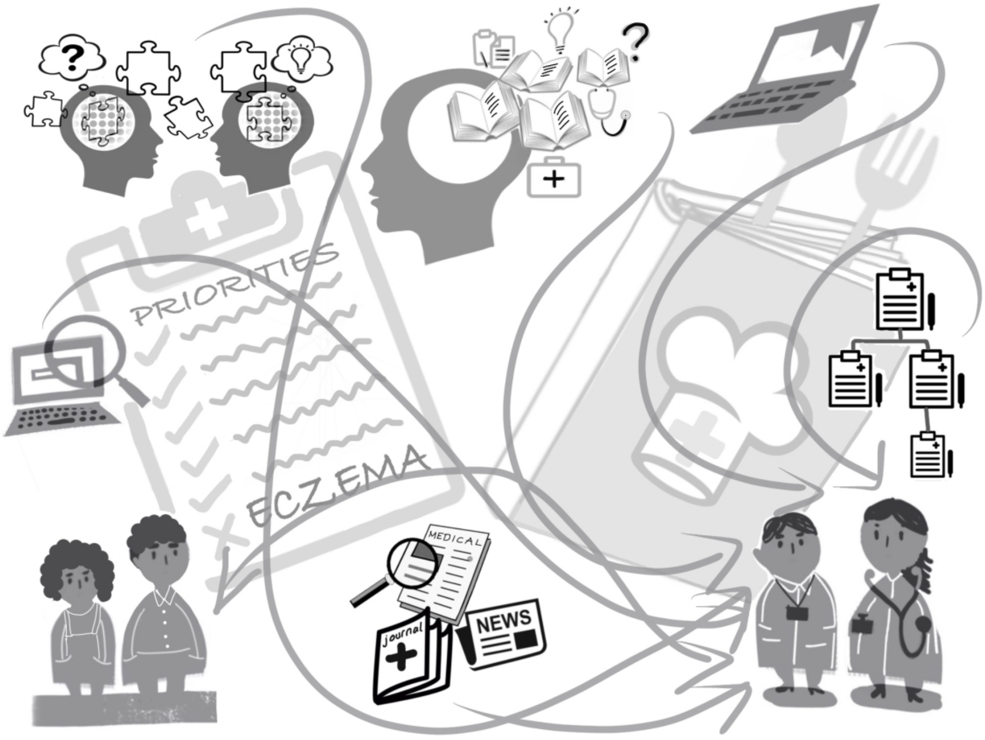

Practitioner eczema mindline

394x295mm (132 x 132 DPI)

BMJ Open Eczema mindlines COREQ 27.11.18

COREQ Statement

Knowledge mobilisation: an exploratory qualitative interview study to confirm and envision modification of lay and practitioner eczema mindlines to improve consultation experiences and self-management in primary care in the United Kingdom

Statement	Page no
**Domain 1: Research team and reflexivity**<br>*Personal Characteristics*<br>1. Interviewer/facilitator Which author/s conducted the interview or focus group?<br>*I conducted all observation and interviews*	5
2. Credentials What were the researcher's credentials? E.g. PhD, MD<br>*DProf, RN*	Title page
3. Occupation What was their occupation at the time of the study?<br>*Professor of Nursing and Health Research*	Title page
4. Gender Was the researcher male or female?<br>*Female*	Title page
5. Experience and training What experience or training did the researcher have?<br>*I have extensive experience in qualitative research.*	Title page
Relationship with participants<br>6. Relationship established Was a relationship established prior to study commencement?<br>*I established rapport with each participant at the time of interview.*	6
7. Participant knowledge of the interviewer<br>What did the participants know about the researcher? e.g. personal goals, reasons for doing the research<br>*I sent participants an information sheet prior to interviews and answered any questions, I reiterated this information at the beginning of each interview.*	6
8. Interviewer characteristics What characteristics were reported about the interviewer/facilitator? e.g. Bias, assumptions, reasons and interests in the research topic<br>*Participants were aware, and it is reported in the manuscript, that I am a Registered Nurse with an interest in how eczema knowledge is developed and shared between patients and clinicians in primary care and that this was a publically funded study.*	5
**Domain 2: study design**<br>*Theoretical framework*<br>9. Methodological orientation and theory<br>What methodological orientation was stated to underpin the study? e.g. grounded theory, discourse analysis, ethnography, phenomenology, content analysis<br>*In the methods section I explain that this is an exploratory qualitative study and that data analysis mainly involved constant comparison.*	5 and 6
Participant selection<br>10. Sampling How were participants selected? e.g. purposive, convenience, consecutive, snowball<br>*I selected participants using maximum variation purposive sampling to ensure a mix of ages, gender and ethnic background.*	5
11. Method of approach How were participants approached? e.g. face-to-face, telephone, mail, email<br>*Practitioners were recruited via the local Clinical Research Network and word of mouth and lay participants though a higher education institute website with mass sharing via word of mouth and media posts*	5
12. Sample size How many participants were in the study?<br>*32 interviews were completed*	5
13. Non-participation How many people refused to participate or dropped out? Reasons?<br>*No participants dropped out.*	NA
Setting<br>14. Setting of data collection Where was the data collected? e.g. home, clinic, workplace<br>*Interview data were collected in workplaces or on the telephone*	6

For peer review only

BMJ Open Eczema mindlines COREQ 27.11.18

16. Description of sample What are the important characteristics of the sample? e.g. demographic data, date<br>Interview participants were sampled by age, gender and ethnicity.	Tables 2 and 3<br>5
Data collection<br>17. Interview guide Were questions, prompts, guides provided by the authors? Was it pilot tested?<br>*A topic guide was used for interviews*	Table 1<br>5
18. Repeat interviews Were repeat interviews carried out? If yes, how many?<br>*No repeat interviews were carried out*	NA
19. Audio/visual recording Did the research use audio or visual recording to collect the data?<br>*Interviews were audio recorded*	5
20. Field notes Were field notes made during and/or after the interview or focus group?<br>*No*	NA
21. Duration What was the duration of the interviews or focus group?<br>*Interviews lasted from 16-55 minutes*	6
22. Data saturation Was data saturation discussed?<br>*Yes*	6
23. Transcripts returned Were transcripts returned to participants for comment and/or correction?<br>*No*	NA
**Domain 3: analysis and findings**<br>*Data analysis*<br>24. Number of data coders How many data coders coded the data?<br>*I analysed the data independently.*	6
25. Description of the coding tree Did authors provide a description of the coding tree?<br>*No*	NA
26. Derivation of themes Were themes identified in advance or derived from the data?<br>*Findings were derived inductively from the data*	7
27. Software What software, if applicable, was used to manage the data?<br>*No*	NA
28. Participant checking Did participants provide feedback on the findings?<br>*No*	NA
Reporting<br>29. Quotations presented Were participant quotations presented to illustrate the themes / findings? Was each quotation identified? e.g. participant number<br>*Quotations are provided and attributed to individuals.*	6-12
30. Data and findings consistent Was there consistency between the data presented and the findings?<br>*Yes.*	6-12
31. Clarity of major themes Were major themes clearly presented in the findings?<br>*Yes.*	6-12
32. Clarity of minor themes Is there a description of diverse cases or discussion of minor themes?<br>*Yes, I present a broad spectrum of participant's views.*	6-12

For peer review only
